# Evaluation of the role of location and distance in recruitment in respondent-driven sampling

**DOI:** 10.1186/1476-072X-10-56

**Published:** 2011-10-18

**Authors:** Nicky McCreesh, Lisa G Johnston, Andrew Copas, Pam Sonnenberg, Janet Seeley, Richard J Hayes, Simon DW Frost, Richard G White

**Affiliations:** 1 Department of Infectious Disease Epidemiology, Faculty of Epidemiology & Population Health, London School of Hygiene and Tropical Medicine, Keppel Street, London, WC1E 7HT, UK; 2 Department of International Health & Development, Center for Global Health Equity, Tulane University School of Public Health & Tropical Medicine, Canal Street, New Orleans, LA 70112, USA; 3 Research Department of Infection and Population Health, University College London, Gower Street, London, WC1E 6BT, UK; 4 MRC/UVRI Uganda Research Unit on AIDS, Entebbe, Uganda; 5 School of International Development, University of East Anglia, Norwich, NR4 7TJ, UK; 6 Department of Veterinary Medicine, University of Cambridge, Madingley Road, CB3 0ES, UK

## Abstract

**Background:**

Respondent-driven sampling(RDS) is an increasingly widely used variant of a link tracing design for recruiting hidden populations. The role of the spatial distribution of the target population has not been robustly examined for RDS. We examine patterns of recruitment by location, and how they may have biased an RDS study findings.

**Methods:**

Total-population data were available on a range of characteristics on a population of 2402 male household-heads from an open cohort of 25 villages in rural Uganda. The locations of households were known a-priori. An RDS survey was carried out in this population, employing current RDS methods of sampling and statistical inference.

**Results:**

There was little heterogeneity in the population by location. Data suggested more distant contacts were less likely to be reported, and therefore recruited, but if reported more distant contacts were as likely as closer contacts to be recruited. There was no evidence that closer proximity to a village meeting place was associated with probability of being recruited, however it was associated with a higher probability of recruiting a larger number of recruits. People living closer to an interview site were more likely to be recruited.

**Conclusions:**

Household location affected the overall probability of recruitment, and the probability of recruitment by a specific recruiter. Patterns of recruitment do not appear to have greatly biased estimates in this study. The observed patterns could result in bias in more geographically heterogeneous populations. Care is required in RDS studies when choosing the network size question and interview site location(s).

## Background

Hidden or hard-to-reach population subgroups, such as sex workers or men who have sex with men, are often key to the spread and maintenance of infectious diseases in human populations [1]. It can be difficult to estimate the prevalence of infection and risk factors in these populations as it may not be possible to obtain a representative sample, either because there may not be an adequate sampling frame or because the groups may be involved with illicit activities or subject to stigma. A variety of convenience sampling techniques are typically used to collect data on these populations [2], however they cannot be used to generate unbiased population-based estimates.

Respondent-driven sampling (RDS)[3] is a variant of a link tracing design that is designed to minimise the bias in estimates of the prevalence of a disease or risk factors in a population. First, a small number of *seeds *are selected by convenience. The seeds are given coupons, usually three, to recruit others from the target population. After being interviewed, the recruits can then themselves become recruiters. Recruits are given incentives both for taking part in the survey and also for recruiting others. This process continues in recruitment 'waves' until the desired sample size is reached or until the distribution of participant characteristics (such as the proportion infected) has become similar between waves (called reaching 'equilibrium' in RDS terminology), ensuring that the final sample is not biased by the choice of seeds. Estimation methods are then applied to account for the non-random sample selection in an attempt to generate unbiased estimates for the target population. Two main estimation methods are used: RDS-1, which accounts for patterns of recruitment between subgroups and the average number of other members of the target group recruiters know (the 'network size') in each subgroup [4,5]; and RDS-2, which accounts for network size only [6]. Members of a recruit's network are known as their 'contacts'.

In the majority of RDS studies, recruits are responsible both for making their own way to the interview site and for finding and approaching other potential recruits. Differences between members of the eligible population in their willingness and ability to travel to an interview site, and between recruits in their willingness and ability to travel to recruit others, have the potential to cause bias in the results of RDS studies, and some RDS studies have found that little or no recruitment occurred in certain parts of the target study areas [7-9]. Despite this, to our knowledge no other RDS studies have looked in detail at how the locations of recruits' residencies affected recruitment, perhaps due to the difficulties of collecting such information from members of a hidden population.

**Figure 1:**
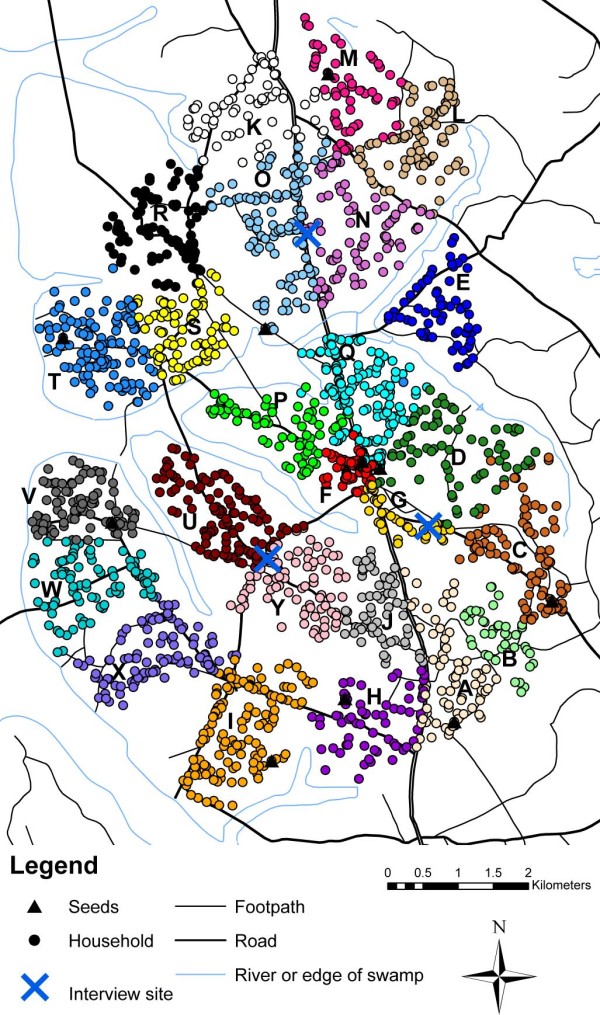
**Map of study area showing location of target population and seed households and RDS interview sites**. Colours are used to represent households in different villages. Each village has been labelled with a letter for confidentiality.

The aim of our RDS study was to evaluate whether RDS could generate representative data on a rural Ugandan population by comparing estimates from an RDS survey with total-population data. The target population for our study consisted of 2402 male household heads living in 25 villages in rural Uganda which make up an ongoing open cohort (the 'General Population Cohort')[10]. The villages cover an area of approximately 38 km^2^. Figure 1 shows a map of the area. Villages are labelled with letters for confidentiality. The three RDS interview sites are shown with blue crosses. Data on the target population were available from annual censuses, questionnaires and blood tests. The full results of the study are given in [11] and McCreesh N, Nadagire Tarsh M, Seeley J, Katongole J, White RG: **Community understanding of Respondent-Driven Sampling in a medical research setting in Uganda: importance for the use of RDS for public health research**, submitted. In brief, both the sample proportions and the RDS-adjusted estimates were representative of the target population in most respects, but younger men, men of higher socioeconomic status, men of unknown HIV status, and men with an unknown number of sexual partners in the previous year were under-represented. There were high levels of homophily (indicating high within-group recruitment) by religion and tribe and in the highest socioeconomic group, however some recruitment occurred between all tribes, religions, and socioeconomic status groups [11].

To our knowledge, this is the first RDS study for which the location of the household of each member of the target population was known a-priori, and therefore the first study to be able to robustly explore how household location and distance to interview site affects RDS recruitment. We examine the distribution of population characteristics by village, patterns of recruitment between villages, distance between recruiters' and their recruits and contacts, the influence on recruitment of which trading centre is closest compared to the influence of which village centre is closest, and whether the locations of the interview sites affected recruitment. We also examine whether the patterns of recruitment we found could have biased the results of our study, and discuss the potential that similar patterns of recruitment would have to cause bias in estimates in other RDS studies.

In this paper men who were offered and accepted coupons to recruit others are called 'recruiters'. The phrases 'distant contact' and 'distant recruit' refer solely to the distance between the men's houses, and do not refer to the closeness of the relationship. Unless stated otherwise, distances were calculated as the shortest straight-line distance between two points (ie 'as the crow flies'). In looking at patterns of recruitment with respect to characteristics, crude RDS sample proportions, and not RDS-adjusted estimates, were used. This was because RDS-adjustment did not improve the estimates of population proportions in our study (see [11]).

## Results

### Distribution of population characteristics and recruitment by village

**Figure 2:**
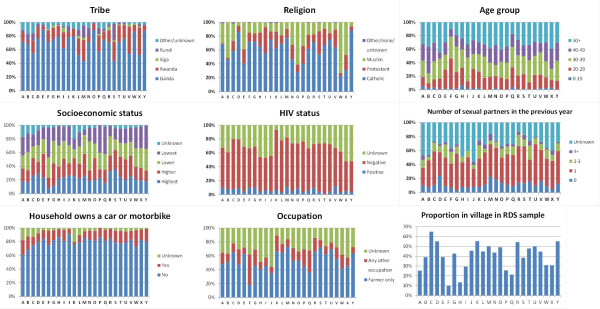
**The distribution of population characteristics and the percent of eligible men in village recruited into the RDS study, by village**.

The percentage of men in each village who were of each tribe, religion, age group, socioeconomic group, HIV status, sexual activity level and occupation and the percentage in each village that lived in a household that owns a car and/or motorcycle are shown in Figure 2. There was little systematic heterogeneity in the population by location, except for the characteristics socio-economic status, tribe and occupation. The percentage of eligible men in each village recruited into the RDS study ranged between 10% (village F) and 65% (village C) (p < 0.001).

**Figure 3:**
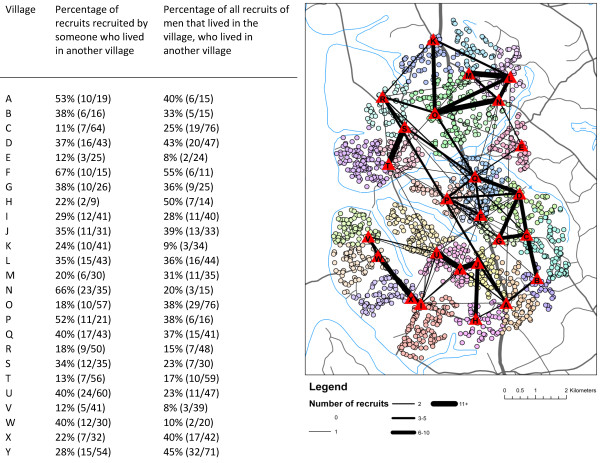
**Number and percentage of recruits recruited between villages**. Triangles show the location of the main village meeting place. The black lines between villages indicate the sum of the number of recruits living in village A (for example) recruited by someone living in village B (for example) plus the number of recruits living in village B recruited by someone living in village A (ie 3 to 5 between village A and village B in this example).

Between 11% (village C) and 67% (village F) of recruits were recruited by someone who lived in another village, and recruits in each village were recruited by people from between two (village H) and eight (village F) villages (Figure 3). A maximum of 23 recruits in any one village were recruited by men who lived in any other one village (men in village U recruited by men in village Y), and a maximum of 27 recruits were recruited in total in either direction between any two villages (village U and village Y). The percentage of all the recruits of men who lived in any one village, who lived in another village, varied between 8% (village E) and 55% (village F).

**Figure 4:**
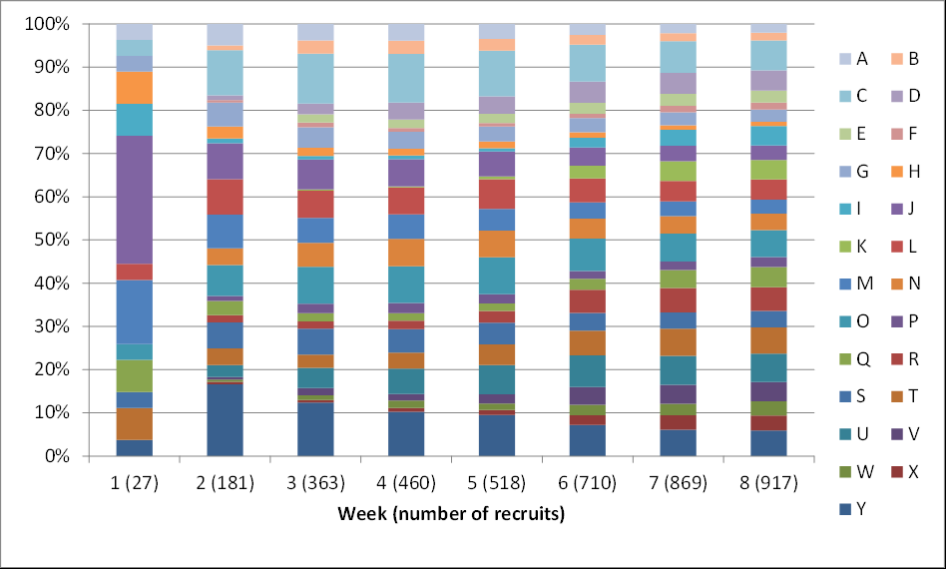
**Percentage of recruits in the RDS sample in each village, by week**. The numbers in brackets are the number of men who had been recruited by the end of that week. Seeds are excluded. All recruitment was ceased by study staff at the end of week eight.

The percentage of men from each village in the RDS sample at the end of each week of recruitment varied markedly during the survey (Figure 4).

The minimum distance along well established paths from the centre of each village to the nearest interview site ranged from 0.00 km (village U) to 3.57 km (village E).

### Trading centres

**Figure 5:**
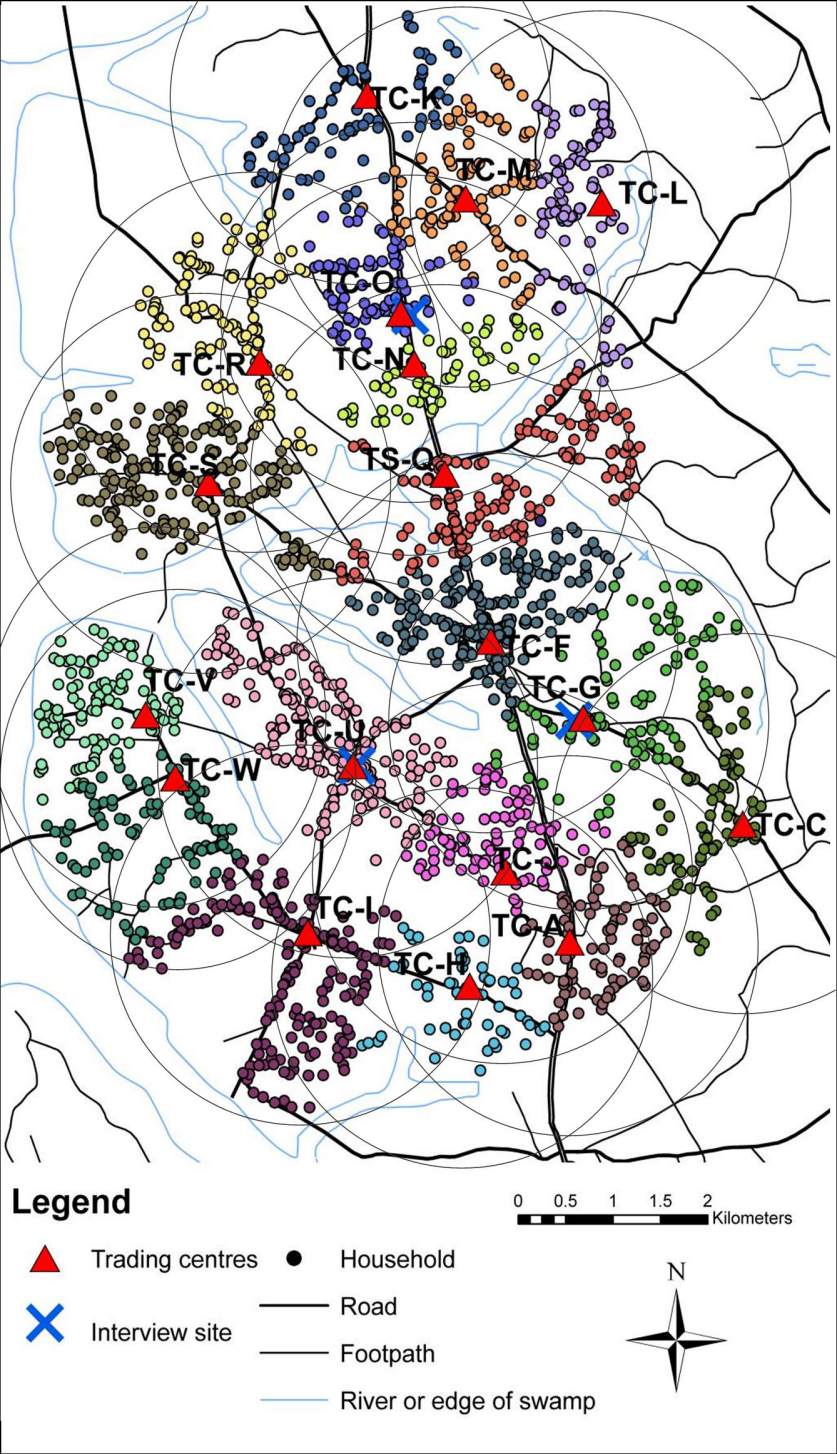
**Map of study area showing location of trading centres**. Colours are used to represent which households were closest to the different trading centres. Trading centres are labelled with the letter of the nearest village. Light grey circles show the area with 2 km of each trading centre.

During analysis of study data, we hypothesised that some recruitment may have occurred at trading centres. Addition data were collected showing that there were 18 trading centres within the study area (red triangles in Figure 5). The percentage of the eligible population who lived closest to each trading centre ranged from 3% for TC-H to 16% for TC-F. Houses in Figure 5 are coloured according to the trading centre to which they were closest. 78.0% of the eligible population lived within 1 km of the nearest trading centre, 21.7% lived between 1-2 km away, and 0.3% lived more than 2 km away. The furthest distance any member of the eligible population lived from a trading centre within the area was 2.07 km.

### Distance between recruits' and recruiters' houses

Additional file 1 shows the locations of recruits' and their recruiters' houses, and illustrates the fact that the majority of men were recruited by someone who lived close to them. Only 7% (66) of recruits were recruited by someone who lived more than 2 km away. Recruits recruited during the first two weeks of the study were more than twice as likely to have been recruited by someone living more than 2 km away, compared to recruits recruited later in the study (13% compared to 6%, p = 0.001). Compared to men recruited by someone who lived less than 2 km away, men recruited by someone who lived more than 2 km away were less likely to report seeing their recruiter daily (42% compared to 70%, p < 0.001), more likely to report that they had known their recruiter for less than 10 years (26% compared to 15%, p = 0.02), and were more likely to have a work relationship with their recruiter (9% compared to 2%, p < 0.001). They were also more likely to have had a longer delay between their recruiter's interview and their own interview. 17% of recruits who lived more than 2 km away from their recruiter were interviewed between one and two weeks after their recruiter, compared to 16% of recruits who lived closer, and 9% were interviewed more than two weeks after, compared to 3% of closer recruits (p = 0.05).

Owning a car and/or motorbike was associated with higher odds of being recruited by someone who lived more than 2 km away (OR = 3.6, p < 0.001). Compared to men who were farmers only, men who had another occupation had higher odds, and men of unknown occupation had lower odds of being recruited by someone who lived more than 2 km away (OR = 2.9 and 0.9 respectively, p = 0.002). Adjusting for occupation, owning a car and/or motorbike was associated with 3.2 times the odds of being recruited by someone who lived more than 2 km away (p < 0.001). Adjusting for car and/or motorbike ownership, compared to men who were farmers only, men who had another occupation had 2.6 times the odds, and men of unknown occupation had 0.9 times the odds, of being recruited by someone who lived more than 2 km away (p = 0.005). There was no evidence that tribe, religion, age group, HIV status, socioeconomic status, sexual activity level or household bicycle ownership were associated with being recruited by someone who lived more than 2 km away.

There was a higher mean reported network size among recruits who owned a car or motorbike of 13.8, compared to 11.9 among recruits who did not own a car or motorbike (p = 0.005). Mean reported network size among recruits with an occupation other than farming was 12.7 compared to 11.9 among recruits who reported farming only (p = 0.1). Mean reported network size among men recruited by someone who lived more than 2 km away was 12.6 compared to 12.0 among men recruited by someone who lived closer (p = 0.5).

There was no evidence for any difference in the ability to recruit reported contacts by distance between recruits' and recruiters' houses. The mean ratio of number of recruits, to number of reported contacts reported in the first interview was 0.285 for recruits/contacts who lived less than 1 km away from the person naming them as a contact and 0.275 for recruits who lived more than 1 km away (p = 0.7). However, recruits who lived closer to the recruiter were more likely to be named as a contact by their recruiter at the first interview. 33% of recruits who were recruited by someone who lived less than 1 km away were named as a contact by their recruiter at the first interview, compared to only 19% of recruits who lived further away (p < 0.001). This suggests that of all contacts known to recruiters, more distant contacts were more likely to be under-reported compared to closer contacts. Together these two findings suggest that of all contacts known to recruiters, more distant contacts may have been under-reported, and under-recruited, compared to closer contacts.

### Distance from village centres and trading centres

In unadjusted analysis, living more than 1 km away from the nearest trading centre was associated with 0.53 times the odds of a recruiter recruiting two or three recruits (p = 0.001) compared to recruiting one or no recruits, and living more than 1 km away from the nearest village centre was associated with 0.59 times the odds (p = 0.04). After controlling for the other measure of distance, living more than 1 km away from the nearest trading centre was associated with 0.57 times the odds (p = 0.01) and there was no evidence that living more than 1 km from the nearest village centre was associated (OR = 0.83, p = 0.5).

71% of men were recruited by someone with the same (closest) trading centre. 71% of men were recruited by someone who lived in the same village. 13% of men were recruited by someone who lived in a different village, with the same closest trading centre. 12% were recruited by someone who lived in the same village, with a different closest trading centre. There was no evidence for a difference between the two percentages (p = 0.5), suggesting that both village membership and nearest trading centre influenced patterns of recruitment. Amongst men recruited by someone who lived more than 1 km away, 18% were recruited by someone who lived in a different village, with the same closest trading centre and 16% were recruited by someone who lived in the same village, with a different closest trading centre. Again, there was no evidence for a difference (p = 0.6).

In unadjusted analysis, living more than 1 km away from the nearest trading centre was associated with 1.17 times the odds of having been recruited into RDS (p = 0.1). After controlling for socioeconomic status, it was associated with 1.14 times the odds (p = 0.2). Living more than 1 km away from the nearest village centre was crudely associated with 1.15 times the odds of having been recruited into RDS (p = 0.03). After controlling for socioeconomic status, it was associated with 1.12 times the odds (p = 0.08).

### Distance from the nearest interview site

**Figure 6:**
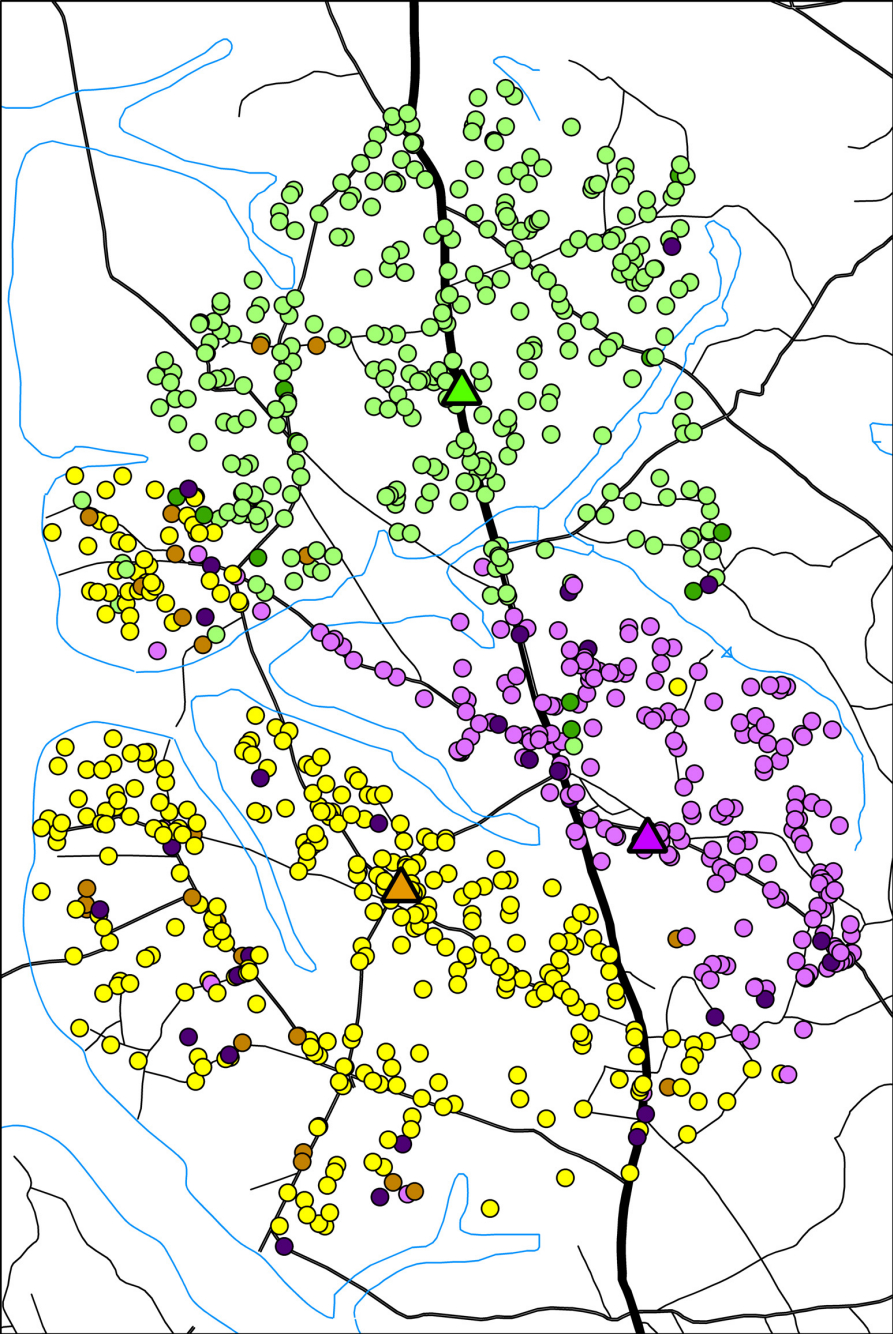
**Recruit interview sites**. The triangles show the location of the interview sites. The circles show recruits' houses and their colour indicates the site at which the recruit was interviewed. Darker shades indicate that the recruit was interviewed at a different site from their recruiter.

7% (65) of recruits were interviewed at a different interview site from their recruiter. 17% (155) of recruits were not interviewed at their nearest interview site (measured as the direct distance) (Figure 6).

Amongst all men in the target population, living more than 1 km away from the nearest interview site was associated with 0.57 times the odds of being recruited into RDS (p < 0.001). Men who lived more than 1 km away from the nearest interview site had a mean reported network size of 11.3, compared to 11.6 for men who lived less than 1 km away (p = 0.5). The difference in recruitment probability by distance is therefore unlikely to have been due to a difference in network size. For men over the age of 50 years, living more than 1 km away was associated with 0.60 times the odds of recruitment (p = 0.01). For men under the age of 50 years, it was associated with 0.56 times the odds (p < 0.001). There was no evidence of any interaction between age and distance (p = 0.8). Amongst the 1066 men in the target population who were known by us to have received a coupon (either because we were told so by a recruiter at their follow-up interview or because they were a recruit), living more than 1 km away from the nearest interview site was associated with 0.67 times the odds of being recruited into RDS (p = 0.1).

Amongst all men in the target population, for men who lived in a household without a car or motorbike, living more than 1 km away was associated with 0.53 times the odds of recruitment (p < 0.001). For men who lived in a household with a car and/or motorbike, it was associated with 0.54 times the odds (p = 0.04). There was no evidence of any interaction between car/motorbike ownership and distance (p = 0.97).

Amongst all men in the target population, after controlling for tribe, having an occupation other than farming was associated with 0.76 times the odds of living more than the population average distance away from the nearest interview site (1.85 km) (p = 0.02). After controlling for occupation, being a member of the Nyanrwanda/kole, Kiga, and Rundi tribes was associated with 1.67, 3.77, 1.58 times the odds respectively, and being a member of an unknown tribe was associated with 1.21 times the odds, of living more than the population average distance away (p < 0.0001), compared to members of the Ganda tribe (the largest tribe in the study area). There was no evidence that any of the other characteristics we explored were associated with distance from the nearest interview site.

The mean distance to the nearest interview site decreased from 1.92 km for recruits interviewed in the first week of the study to 1.49 km for recruits interviewed in week three, and then increased to 2.10 km for recruits interviewed in week seven before falling slightly to 2.07 km for recruits interviewed in week eight (p < 0.001).

## Discussion

In contrast to some RDS studies which reported that little or no recruitment occurred in some parts of the study areas [7-9], all 25 villages were represented in the RDS sample. This may have been due to the relatively small study area and large sampling fraction in our study, and to the absence of any major structural or social barriers or bottlenecks in the population. There was a large difference in the percentage of each village recruited into the RDS study however, ranging from 10% to 64%. There was little heterogeneity in the distribution of key population characteristics of interest by location. Our results suggest that more distant contacts were less likely to reported by recruits and therefore less likely to be recruited. However, if reported, more distant contacts were as likely to be recruited as closer contacts. There was no evidence that living closer to the nearest trading centre was associated with probability of being recruited, but it was associated with a higher probability of recruiting a larger number of recruits. People living closer to an interview site were more likely to be recruited.

The main limitation of our study is that, due to the fact that only the major paths (paths that can be travelled by motorbike) in the area have been mapped, all distances were calculated as the direct straight-line distance. Although the distances given here will not correspond exactly with the distances people walk, for distances within villages and between villages with no major obstacles between them (eg swamps), the relative distances are likely to be approximately correct. This is because most of the study area consists of small fields with a network of paths between them. Inaccuracies in the absolute and relative sizes of larger distances may have reduced or hidden true differences in mean distances or proportions by distance, however this is unlikely to have introduced any systematic bias into the study and is therefore unlikely to be responsible for the results we found. It should be noted, however, that in the majority of RDS studies direct straight-line distance would not be an appropriate measure of distance and other measures should be considered.

Our results suggest that more distant contacts may have been under-recruited. Provided that this occurred equally across all groups of a characteristic, this should not have biased either the RDS sample proportions or the RDS-estimates, although it may have increased the number of waves required to reach equilibrium. However, if members of a particular group were more likely to be recruited by more distant contacts (for instance, a group was more mobile), then that group may be over-represented in the RDS sample. Provided that the assumptions behind RDS theory are met, if that group under-reports their own more distant contacts less than other groups do, then the RDS-1 and RDS-2 estimation methods should be able to reduce this bias. If they do not, then the RDS estimators will not reduce this bias. In our study, owning a car or motorbike and having an occupation other than farming were associated with increased odds of having been recruited by someone living more than 2 km away. Mean reported network size was slightly higher among recruits from these groups. This suggests that RDS estimators may have been able to correct at least some of any bias caused by over-recruitment of these groups by more distant contacts. The differences in mean network size are small however, and therefore RDS-adjustments may not fully correct any bias. Unfortunately, it was not possible to test this directly using our data because this was obscured by another recruitment bias, the under-recruitment of higher socioeconomic status men (see [11] and McCreesh N, Nadagire Tarsh M, Seeley J, Katongole J, White RG: **Community understanding of Respondent-Driven Sampling in a medical research setting in Uganda: importance for the use of RDS for public health research**, submitted).

A priori we assumed there would be strong within village recruitment. However in this setting, villages are primarily administrative areas and do not, in general, affect day to day life for the residents. During post-hoc analysis therefore, it was hypothesised that the observed strong tendency for recruits to recruit from their own village may be explained by a tendency for recruiters to recruit people who lived to close to them, and/or a tendency for recruiters to recruit people who lived closest to the same trading centre. The proportion of men who were recruited by someone who lived in the same village but had a different closest trading centre was similar to the proportion of men who were recruited by someone who lived in a different village but had the same closest trading centre, both when all recruits were considered and when only recruits recruited by someone who lived more than 1 km away were considered. This suggests that both village membership and closest trading centre influenced patterns of recruitment.

The most likely explanation for the surprising result that people who lived more than 1 km away from the nearest village centre were more likely to have been in the RDS sample than people who lived closer to the nearest village centre, was residual confounding by socioeconomic status. Men of higher socioeconomic status were more likely to live within 1 km of the nearest village centre, and were less likely to have participated in the RDS study (see [11]). Controlling for socioeconomic status slightly reduced the odds ratio for participation in RDS for men who lived more than 1 km away compared to men who lived less than 1 km away. It is likely that the odds ratio would have been further reduced if our measure of socioeconomic status better approximated the element of socioeconomic status associated with both place of residence and participation in RDS.

Three interview sites were used in our study and the path distance from the centre of any study village to the nearest interview site was no more than 3.57 km. Nevertheless, the mean distance to the nearest interview site was slightly further for non-recruits compared to recruits, and further still for men who were recorded as having been given a coupon by a recruiter but who did not become recruits, suggesting that men who lived further from the nearest interview site were less likely to be recruited.

There are two ways that an under-recruitment of members of the eligible population who live further from the nearest interview site could bias estimates. First, if the distribution of characteristics among more distant people is different from the distribution among closer people. This was the case for occupation and tribe in our study. Unfortunately, due to other biases affecting recruitment by socioeconomic status (and therefore occupation), it was not possible to tell if an under-recruitment of more distant men biased the estimates of occupation. The sample proportions and RDS-estimates for tribe were close to the true population proportions [11], suggesting that the under-recruitment of more distant men did not bias the estimates for tribe. The same tribes that had a higher proportion of more distant men also had a higher proportion of lower socioeconomic status men however, and therefore the bias towards an over-recruitment of lower socioeconomic status men may be masking any bias caused by the under recruitment of more distant men. Second, under-recruitment of more distant people could bias estimates if the under-recruitment affects some groups more than others. This was tested for older men and men who did not own a car or motorbike in our study, but there was no evidence it caused biased estimates. However, this does not mean that in other studies with more geographic heterogeneity, under-recruitment of more distant people would not lead to bias.

It should be noted that this study was carried out in a non-hidden population who were unlikely to be concerned about the possibility of acquaintances seeing them visit an interview site. In RDS studies of stigmatised groups, a fear of being 'outed' as a member of the group may lead to an under-recruitment of people who live or work close to the interview site, in addition to any under-recruitment of more distant people. Having multiple interview sites could potentially reduce this problem.

Our study was carried out in a rural area where the majority of people work as farmers. This is in contrast to many RDS studies which are carried out in towns or cities. In these settings, travel times using available transport and travel costs may be more important than distances. For instance, one urban RDS study recruited few men from a certain area of a city. They suggested that this was due to the fact that the neighbourhood was geographically isolated by highway systems and poor public transport access [8]. In addition to this, individuals in urban areas may have multiple sites they visit regularly in addition to their homes (eg work places), and the locations of all these sites may influence recruitment. Overall patterns of recruitment may therefore be more complex in urban settings. They are nevertheless likely to include many of the same basic patterns (eg recruiters recruiting contacts who are geographically more accessible to them in some way), and so the conclusions of this study may be generalisable to RDS studies of urban populations. Social geography may also need to be considered in addition to physical geography in many populations however, for instance if certain groups in the target population have little social contact with other, geographically close, groups. Urban populations may also be more geographically heterogeneous than our rural population, meaning that patterns of recruitment by location and distance may have a greater potential to result in bias.

## Conclusions

Our findings suggest that two things need to be considered when designing RDS studies. First, care needs to be taken when deciding on the main network size question. Any tendency for certain groups to be recruited more or less by more distant contacts may result in that group being over or underrepresented in the RDS sample. To enable RDS-adjustment to correct for this adequately, the chosen network size measure must accurately reflect recruitment probability both among people who travel little outside the area they live in, and among people who travel more widely throughout the study area. Second, care should be taken when deciding where to locate interview sites, and whether more than one is required. Even with the relatively small largest distance between any member of the eligible population and the nearest interview site in this study, more distant members of the eligible population were less likely to be recruited. This tendency has the potential to cause bias in more geographically heterogeneous populations.

## Methods

### RDS study

Each year, after obtaining consent, a total-population household census and an individual questionnaire and HIV-1 serosurvey are administered to the entire General Population cohort. The target population consisted of 2402 men who were recorded as a male head of a household within these villages between February 2009 and January 2010. The percent of the target population living in each village ranged from 2% (in village B) to 9% (in village Q).

Ten seeds were purposively selected from the target population to vary in age, tribe, and village. Villages A,C,F,H,I,M,O,Q,T and V each contained a seed. All seeds successfully recruited men into the study, with the number of waves originating from each seed ranging from 3 to 16. Nine hundred and twenty seven men (including seeds) were recruited into the study in total over a period of 54 days (8th March - 30th April 2010).

Seeds and recruits were offered incentives for participation and recruitment, either soap, salt or school books to the value of ~$1US. One incentive was offered for completing the first interview and another for each person successfully recruited.

The main measure of recruits' network sizes was determined from their response to the question: "How many men do you know who (i) were head of a household in the last 12 months in any of the MRC villages, and (ii) you know them and they know you, and (iii) you have seen them in the past week". Recruits were also asked to recall the names and/or other demographic characteristics of each individual member of their network who may be eligible. These details were used by the interviewer to search the general population cohort database (containing details of all men known to the MRC irrespective of eligibility for the general population cohort or RDS) and attempt unique identification. If the man was positively identified as someone in the general population cohort database, this was recorded. 83% of all recorded contacts were identified and 76% were identified and eligible. These identified and eligible men make up the recruits' contacts in this paper.

Unless stated otherwise, all distances were calculated as the direct straight-line distance ('as the crow flies') using Hawth's Analysis tools for ArcGIS [12] extension for ArcGIS [13].

### Data sources and analysis

Data were available on the tribe, religion, age, socio-economic status, number of sexual partners in the previous year (sexual activity level), and HIV status of the target population (for details see [11]). Household socioeconomic status was calculated using principal components analysis from household ownership of 22 items recorded during an annual census (December 2008-October 2009) and categorised into quartiles based on the status of all households in the general population cohort villages. Data on household ownership of cars, motorbikes, and bicycles were available from a household census carried out between November 2008 and September 2009. Data on occupation were taken from the most recent general population cohort round (carried out between December 2009 and October 2010), or if this was unavailable, from the previous survey round (December 2008 - October 2009).

The minimum distances between the centres of all villages and the nearest interview site along well defined paths were calculated.

The locations of all trading centres (areas with shops) within the study area were recorded. For each member of the target population, the nearest trading centre and the distance to the nearest trading centre were determined. Distances were measured as the direct distance. Where the nearest trading centre using direct distances would clearly not be the nearest trading centre travelling along paths (ie there was a swamp in the way), the nearest trading centre along paths was determined by looking at a map of the area. This trading centre was taken as the nearest trading centre, and the direct distance to it was used as the distance to the nearest trading centre. Trading centres are labelled with the letter of the village they are in.

The association between the characteristics tribe, religion, age group, socioeconomic status, HIV status, sexual activity level, household ownership of a bicycle, household ownership of a car and/or motorbike and occupation and having being recruited by someone who lived more than 2 km away was explored using logistic regression. Characteristics were included in the final model if p < 0.05.

The crude odds ratio of a recruiter recruiting two or three recruits (as opposed to zero or one recruits) was calculated for men living more than 1 km away from the nearest trading centre compared to men living closer, and for men living more than 1 km away from the nearest village centre, compared to men living closer. Adjusted odds ratios were also calculated, adjusting for the other measure of distance (living more or less than 1 km away from the nearest village centre or living more or less than 1 km away from the nearest trading centre).

The percentage of recruits who were recruited by someone with the same nearest village centre and a different nearest trading centre, and the percentage who were recruited by someone with the same nearest trading centre but a different nearest village centre were calculated. McNemar's test for matched pairs was used to assess the evidence for a difference between the two percentages. This was repeated for recruits who had been recruited by someone living more than 1 km away only.

Crude and adjusted odds ratios were calculated for the odds of having being recruited into the RDS sample for men living more than 1 km away from the nearest trading centre compared to men who lived closer, and for men living more than 1 km away from the nearest village centre compared to men who lived closer. Adjusted odds ratios adjusted for socioeconomic status (categorised into the groups highest, higher, lower, lowest and unknown).

The association between the characteristics tribe, religion, age group, socioeconomic status, HIV status, sexual activity level, household ownership of a car and/or motorbike and occupation and living more than the population average distance away from the nearest interview site was explored using logistic regression. Characteristics were included in the final model if p < 0.05.

### Ethical approval

The Science and Ethics Committee of the Uganda Virus Research Institute (GC/l27109108), the Uganda National Council for Science and Technology (SS2278) and the London School of Hygiene and Tropical Medicine Ethics Committee (5585) gave ethical approval for the study.

## Competing interests

The authors declare that they have no competing interests.

## Authors' contributions

RGW and NM designed the research with contributions from all authors. RGW supervised the data collection. NM collected the data on trading centres. NW analyzed the data and wrote the first draft of the paper with RGW. All authors contributed to and approved the manuscript.

## Supplementary Material

Additional file 1 **Animation showing recruitment over time, and the locations of recruits' and their recruiters' houses**. Grey circles represent target population households, stars represent interview sites, triangles represent seeds, and arrows indicate recruitment during that day. Different coloured arrows indicate recruitment originating from different seeds. Click here for file
